# EDITORIAL COMMENT: LAPAROSCOPIC - ASSISTED TRANSPYELIC RIGID NEPHROSCOPY - SIMPLE ALTER-NATIVE WHEN FLEXIBLE URETEROSCOPY IS NOT AVAILABLE

**DOI:** 10.1590/S1677-5538.IBJU.2014.0588.1

**Published:** 2016

**Authors:** David J. Hernandez

**Affiliations:** 1 USF Urology Clinic South Urologic Malignancies; 2 Department of Urology, Tampa General Hospital, Tampa, FL, USA

Percutaneous nephrolithotomy is generally considered the first choice for the treatment of large upper urinary tract stones. However, laparoscopic stone surgery with or without robotic assistance is a viable alternative especially in cases with aberrant anatomy such as ectopic or malrotated kidneys (1–3). A recent meta-analysis found several advantages of the laparoscopic approach, especially reduced blood loss, higher stone free rate and fewer secondary procedures (3).

Tobias-Machado et al (4) present a video on laparoscopic assisted rigid nephroscopy performed via a transpyelic approach for removal of stones in 2 cases with difficult anatomy. The authors are to be commended for their excellent surgical technique and description. However, using a rigid nephroscope would very rarely be necessary in most urologic centers in the US where flexible cystoscopes and ureteroscopes are nearly always available and preferred to avoid limitations due to angulation. When I have performed robotic pyelolithotomy (typically at concominant robotic pyeloplasty), my preference has been to use a flexible cystoscope.

*David J. Hernandez, MD* *Assistant Professor of Urology* *Director, USF Urology Clinic South* *Urologic Malignancies, Robotic Surgery, BPH & Urolithiasis* *Chief of Urology, Tampa General Hospital* *2 Tampa General Circle, STC 6th floor* *Tampa, FL 33606, USA* *Fax: + 1 813 259-8645* *E-mail: dhernan3@health.usf.edu*
